# Bioviolence: Preventing Biological Terror and Crime

**DOI:** 10.3201/eid1402.071381

**Published:** 2008-02

**Authors:** Richard A. Goodman

**Affiliations:** * Centers for Disease Control and Prevention, Atlanta, Georgia, USA

Even before the anthrax attacks in 2001, public health agencies and partner sectors had begun intensifying efforts to detect and respond to the specter of biologic agents used as instruments of terror. The events in 2001 highlighted the substantial preparedness gaps and needs in multiple dimensions, particularly the requirements for coordinating the work of public health and law enforcement, sectors that operate under different jurisdictional configurations and legal regimes. This book is written by a law professor who begins by positing the thesis that humanity is vulnerable to bioterrorism because current international legal regimes are inadequate to support preventive policies. The author may thus be overly ambitious by attempting to cover this topic on a global scale, rather than through the prism of 1 or a few governance systems.

This book may be particularly helpful to persons who want to learn more about basic concepts regarding the methods of bioterrorism. For example, the second chapter provides an overview and description of biologic agents identified as candidates for use by terrorists, and the third chapter presents a synopsis of historical milestones in the use of bioweapons. The second part of the book offers a conceptual treatment of the author’s beliefs about factors accounting for the global failure to effectively confront the threat of biologic agents by multiple actors, and combines this with a focused discussion of 4 categories of measures to reduce bioterrorism. These categories are interdiction (a practically framed summary), denial of access to methods of bioterrorism, preparedness (i.e., detection and response), and nonproliferation regimens. The author concludes with a call for the establishment of “a global governance architecture for preventing bioviolence.”

The book’s utility for practical applications seems constrained, in part, by a limitation common to single-authored books on topics with myriad and complex technical dimensions. In particular, examining bioterrorism must take into account the convergence of numerous and complex fields, including forensic and laboratory sciences, public health, law enforcement, and behavioral sciences, to name only a few. In addition, although some chapters provide information helpful for shaping readers’ understanding of particular issues, in many instances the text falls short of being practically relevant. For example, within the chapter on public health preparedness, the author devotes only 3 paragraphs to the critically important issue of “law enforcement–public health cooperation,” which, since the 2001 anthrax attacks, has been the focus of several major initiatives within the United States.

An additional point is that the author appears to be coining a new term, *bioviolence* (“...the infliction of harm by the intentional manipulation of living micro-organisms or their natural products for hostile purposes”), for which he also provides a rationale. Yet to be determined is whether this term truly is helpful or possibly confusing because of the already well-established lexicon and conceptions surrounding bioterrorism. On balance, however, this book can be recommended because it helps to address a void in the literature, particularly in relation to concepts of preventing bioterrorism, and because it represents another step toward establishing the multidimensional knowledge base necessary to enhance preparedness.

